# The Psycho-Social Impact of Human-Animal Interactions

**DOI:** 10.3390/ijerph17113964

**Published:** 2020-06-03

**Authors:** Aubrey H. Fine

**Affiliations:** Department of Education, California State Polytechnic University, Pomona, CA 91768, USA; ahfine@cpp.edu

When I was asked by the Journal to guest edit a special issue on the psychosocial impact of human-animal interactions, I was honored to accept the invitation. Over the past year, it has been a privilege to be able to spearhead an issue with this tremendous diversity of the articles incorporated. The field of anthrozoology has evolved over the past several decades into a multi-disciplinary interest studying human animal connections. The discipline was established in the early 1980′s, after the pioneer research highlighting the health benefits of humans interacting with animals. As a result of these findings, a robust research agenda was initiated investigating the value of these interactions on numerous scholarly topics. There was not only an awakening towards investigating these topics, but also a growth in establishing numerous academic curriculums worldwide on anthrozoology, as well as the initiation of a couple of journals to support this evolving field. Over the course of the last 20 years, we have seen an influx of more scholarly papers studying the impact of these interactions, not only on the well-being of humans, but also on the animals themselves. Our relationships with non-human animals are now being examined more extensively and comprehensively. This special issue represents a collection of articles that will enhance the body of literature in this field. 

The field of human-animal interactions still continues to be in need of stronger research to document the importance of human-animal interactions. The media has at times tainted the public’s understanding and has unfortunately sensationalized the outcomes. Many researchers, including this author, believe that solid research and its findings will elevate the status of the field of anthrozoology, and provide those who are skeptical with stronger evidence to follow.

Science, at times, cannot always capture the true essence of some of the outcomes that are discovered, but it is the role of researchers to help build a better understanding. Albert Einstein once insinuated that “*everything that can be counted does not necessarily count; and that everything that counts cannot necessarily be counted.”* Although the editor has always admired this quotation by Einstein that exemplifies some of the uncertainties in life, he believes that the field needs a more rigorous platform of research that will clearly demonstrate the importance of human-animal interactions. With better science, the field of anthrozoology will be able to have a stronger impact on public policy as it pertains to global human-animal interactions. It is imperative that we recognize that good science should serve as the impetus to developing viable policies that impact us all. 

This issue on the psychosocial impact of human-animal interactions incorporates diverse articles including many topics focusing on animal-assisted interventions, the importance of companions animals in our society, and an article helping identify critical elements in understanding the link between animal cruelty and family violence. More specifically, the readers will be able to review research from numerous interdisciplinary scholars discussing the importance of the One Health movement. Within this issue, there are two papers specifically written on One Health. In one of the papers, the authors contend that careful attention must be exercised to avoid a trade-off between the well-being and health of animals versus the contentment of humans. Both humans and animals should benefit in their participation in animal-assisted interventions. A couple of articles within this special issue investigate the roles of animals in improving the well-being of healthcare providers while in the work setting. This topic has significant interest, since animal-assisted interventions can also have a significant impact on the morale and productivity of staff in healthcare settings. These visits by various species of animals could have far-reaching effects impacting the emotional well-being of many individuals.

There are also a few articles highlighting the importance of companion animal ownership and promoting the quality of life of the elderly, as well as those living in urban environments. Finally, the edition consists of a wide array of articles pertaining to animal-assisted interventions. Topics include the role and impact of animal assisted interventions (AAI) with college students, the value of equine-assisted interventions, a discussion on future directions in the field of AAI, and an article investigating the impact of animal-assisted intervention programs for children who have been exposed to gender-based violence. These are merely some of the topics and papers integrated within this special issue. More specifically, I welcome you to review all of the various published papers within the entire edition [1,2,3,4,5,6,7,8,9,10,11,12,13,14,15,16,17,18,19,20,21]. Table 1 summarizes all the articles and highlights the major findings in each paper. The research and outcomes should foster intellectual and scholarly discussions on all of these important topics. 

On numerous occasions, I have noted that, over the past 50 years, science seems to be attempting to document what many lay people have believed intuitively: surrounding oneself with animals can be good, not only for our own well-being, but for the communities that we live in. Having said that, it is also important to appreciate the challenges that presently exist in our society, where our co-existence with other nonhuman animals continues to have negative outcomes. We must remain cognizant at all times to the intricate complexities of these relationships and the impactful negative effects that result from the creation of a non-harmonious environment. Special editions, such as this one, that incorporate peer-reviewed scholarly articles contribute to a better understanding. Hopefully, this edition will support some of your endeavors in bridging scientific findings to enhance and foster more optimal human-animal interactions.

## Figures and Tables

**Table 1:** Summary of Articles Submitted in the Special Issue “The Psycho-Social Impact of Human-Animal Interactions”.

**Author(s)**	**Topic**	**Major Findings**
Jegatheesan et al. [1]	The Link between animal cruelty and family violence	The Link explained through the Bronfenbrenner’s bioecological systems model.
Schroeder et al. [2]	EAI and childhood obesity	Children perceived the treatment acceptable and enjoyable.
Wright et al. [3]	Companion animals and quality of life of gay and bisexual men with prostate cancer	Pets may be a stressor for gay and bisexual men following treatment.
Ortmeyer and Robey [4]	Foster dogs and older veterans	Fostering a dog can improve physical activity, health, and QOL in older veterans.
Muela et al. [5]	AAI and children exposed to gender-based violence	Children showed a reduction in internalizing symptoms and in symptoms associated with PTSD.
Kogan et al. [6]	Veterinarians’ views and experiences with dog breeds, dog aggression, and breed-specific laws	Most feel that banning an entire dog breed is not an effective way to ensure human safety.
Machová et al. [7]	CAT and well-being in nurses	Reduction of cortisol levels with CAT in nurses at high risk of stress.
Krouzecky et al. [8]	Dog companionship and stressful situations	Dog owners assessed daily stressors to be more stressful than non-dog owners did.
Mičková et al. [9]	Dog ownership and health and activity of older adults	Dog ownership may affect the overall physical activity and health of older adults.
Pendry, Kuzara, and Gee [10]	University-based Animal Visitation Programs and student responsiveness	Evidence-based content presentations with HAI were associated with higher levels of enjoyment, perceived usefulness, and likelihood of recommendation.
Gee et al. [11]	Health benefits of live fish	Greater perceptions of relaxation and mood, and less anxiety during or after viewing live fish.
Wagner, Lang, and Hediger [12]	Cats in psychiatric wards and their impact on patients and staff	Positive attitudes of inpatients and staff members toward ward cats.
Rodrigo-Claverol et al. [13]	AAI and pain perception in geriatric patients	AAT led to an additional reduction in pain perception and pain-induced insomnia in individuals with higher baseline severity.
Grandin [14]	Horses and ASD	Working with horses may be a beneficial intervention for youth with ASD.
Wong, Yu, and Ngai [15]	Companion animals and human well-being in Hong Kong	Positive impacts of pet ownership on the well-being of owners may be limited by cramped living space and limited pet ownership.
Carr et al. [16]	Dog ownership and its impact on people with chronic low back pain	Dog owners reported fewer depression and anxiety symptoms, more social ties than non-dog owners, and improved well-being.
Machová et al. [17]	AAT and long-term hospitalizations	AAT did not affect physiological parameters, but it exerted a significant effect on the psychological well-being of the patients.
Fine, Beck, and Ng [18]	The State of AAI	Overview of the history of AAI, current state of animal welfare, public policy regarding AAI, and AAI’s future trajectory.
Lerner [19]	Comprehensive human-animal approach to evaluating AAIs	The modified role and species version of the capabilities approach could evaluate AAIs.
Menna et al. [20]	AAIs and the One Health approach	AAIs should be evaluated systemically as a network within a process, in which every component interacts with, and influences other components.
Hediger, Meisser, and Zinsstag [21]	A One Health framework for AAIs	A One Health study design is necessary to ensure that a tradeoff in health of animals is prevented, and that a synergistic benefit can be achieved.

## References

[1] Jegatheesan B., Enders-Slegers M.-J., Ormerod E., Boyden P. (2020). Understanding the Link between Animal Cruelty and Family Violence: The Bioecological Systems Model. Int. J. Environ. Res. Public Health.

[2] Schroeder K., Van Allen J., Dhurandhar E., Lancaster B., Heidari Z., Cazenave K., Boone D., Erdman P. (2019). Riding into Health: A Case Study on an Equine-Assisted Childhood Obesity Intervention. Int. J. Environ. Res. Public Health.

[3] Wright M.M., Schreiner P., Rosser B.R.S., Polter E.J., Mitteldorf D., West W., Ross M.W. (2019). The Influence of Companion Animals on Quality of Life of Gay and Bisexual Men Diagnosed with Prostate Cancer. Int. J. Environ. Res. Public Health.

[4] Ortmeyer H.K., Robey L.C. (2019). Companion Dog Foster Caregiver Program for Older Veterans at the VA Maryland Health Care System: A Feasibility Study. Int. J. Environ. Res. Public Health.

[5] Muela A., Azpiroz J., Calzada N., Soroa G., Aritzeta A. (2019). Leaving A Mark, An Animal-Assisted Intervention Programme for Children Who Have Been Exposed to Gender-Based Violence: A Pilot Study. Int. J. Environ. Res. Public Health.

[6] Kogan L.R., Schoenfeld-Tacher R.M., Hellyer P.W., Oxley J.A., Rishniw M. (2019). Small Animal Veterinarians’ Perceptions, Experiences, and Views of Common Dog Breeds, Dog Aggression, and Breed-Specific Laws in the United States. Int. J. Environ. Res. Public Health.

[7] Machová K., Součková M., Procházková R., Vaníčková Z., Mezian K. (2019). Canine-Assisted Therapy Improves Well-Being in Nurses. Int. J. Environ. Res. Public Health.

[8] Krouzecky C., Emmett L., Klaps A., Aden J., Bunina A., Stetina B.U. (2019). And in the Middle of My Chaos There Was You?—Dog Companionship and Its Impact on the Assessment of Stressful Situations. Int. J. Environ. Res. Public Health.

[9] Mičková E., Machová K., Daďová K., Svobodová I. (2019). Does Dog Ownership Affect Physical Activity, Sleep, and Self-Reported Health in Older Adults?. Int. J. Environ. Res. Public Health.

[10] Pendry P., Kuzara S., Gee N.R. (2019). Evaluation of Undergraduate Students’ Responsiveness to a 4-Week University-Based Animal-Assisted Stress Prevention Program. Int. J. Environ. Res. Public Health.

[11] Gee N.R., Reed T., Whiting A., Friedmann E., Snellgrove D., Sloman K.A. (2019). Observing Live Fish Improves Perceptions of Mood, Relaxation and Anxiety, But Does Not Consistently Alter Heart Rate or Heart Rate Variability. Int. J. Environ. Res. Public Health.

[12] Wagner C., Lang U.E., Hediger K. (2019). “There Is a Cat on Our Ward”: Inpatient and Staff Member Attitudes toward and Experiences with Cats in a Psychiatric Ward. Int. J. Environ. Res. Public Health.

[13] Rodrigo-Claverol M., Casanova-Gonzalvo C., Malla-Clua B., Rodrigo-Claverol E., Jové-Naval J., Ortega-Bravo M. (2019). Animal-Assisted Intervention Improves Pain Perception in Polymedicated Geriatric Patients with Chronic Joint Pain: A Clinical Trial. Int. J. Environ. Res. Public Health.

[14] Grandin T. (2019). Case Study: How Horses Helped a Teenager with Autism Make Friends and Learn How to Work. Int. J. Environ. Res. Public Health.

[15] Wong P.W., Yu R.W., Ngai J.T. (2019). Companion Animal Ownership and Human Well-Being in a Metropolis—The Case of Hong Kong. Int. J. Environ. Res. Public Health.

[16] Carr E.C., Wallace J.E., Pater R., Gross D.P. (2019). Evaluating the Relationship between Well-Being and Living with a Dog for People with Chronic Low Back Pain: A Feasibility Study. Int. J. Environ. Res. Public Health.

[17] Machová K., Procházková R., Eretová P., Svobodová I., Kotík I. (2019). Effect of Animal-Assisted Therapy on Patients in the Department of Long-Term Care: A Pilot Study. Int. J. Environ. Res. Public Health.

[18] Fine A.H., Beck A.M., Ng Z. (2019). The State of Animal-Assisted Interventions: Addressing the Contemporary Issues That Will Shape the Future. Int. J. Environ. Res. Public Health.

[19] Lerner H. (2019). A Proposal for a Comprehensive Human–Animal Approach of Evaluation for Animal-Assisted Interventions. Int. J. Environ. Res. Public Health.

[20] Menna L.F., Santaniello A., Todisco M., Amato A., Borrelli L., Scandurra C., Fioretti A. (2019). The Human–Animal Relationship as the Focus of Animal-Assisted Interventions: A One Health Approach. Int. J. Environ. Res. Public Health.

[21] Hediger K., Meisser A., Zinsstag J. (2019). A One Health Research Framework for Animal-Assisted Interventions. Int. J. Environ. Res. Public Health.

